# Pharmacogenetic Association between Allelic Variants of the Autophagy-Related Genes and Anti-Vascular Endothelial Growth Factor Treatment Response in Neovascular Age-Related Macular Degeneration

**DOI:** 10.3390/biomedicines11113079

**Published:** 2023-11-16

**Authors:** Oyuna S. Kozhevnikova, Anzhella Zh. Fursova, Anna S. Derbeneva, Ida F. Nikulich, Vasiliy A. Devyatkin, Nataliya G. Kolosova

**Affiliations:** 1 Federal Research Center Institute of Cytology and Genetics SB RAS, Pr. Lavrentiev, 10, 630090 Novosibirsk, Russia; fursova@bionet.nsc.ru (A.Z.F.); derbeneva@bionet.nsc.ru (A.S.D.); nikulich@bionet.nsc.ru (I.F.N.); devyatkin@bionet.nsc.ru (V.A.D.); kolosova@bionet.nsc.ru (N.G.K.); 2 State Novosibirsk Regional Clinical Hospital, St. Nemirovich-Danchenko, 130, 630087 Novosibirsk, Russia; 3 Department of Ophthalmology, Novosibirsk State Medical University, Pr. Krasny, 52, 630091 Novosibirsk, Russia

**Keywords:** age-related macular degeneration, autophagy, response to anti-VEGF therapy, pharmacogenetics, aflibercept

## Abstract

Background: Age-related macular degeneration (AMD) is the leading cause of late-onset blindness in elderly. The occurrence and development of AMD is a multifactorial complex process where autophagy plays an important role. The first-line drugs for neovascular AMD (nAMD) are inhibitors of VEGF, with up to 30% of patients having an incomplete response to treatment. Genetic factors may influence the response to anti-VEGF therapy and explain treatment outcome variability. We aimed to estimate the role of polymorphic markers of the *MTOR* (rs1064261, rs1057079, rs11121704, rs2295080), *SQSTM1* (rs10277), *ULK1* (rs11246867, rs3088051), *MAP1LC3A* (rs73105013) and *ATG5* (rs573775) genes in the development of nAMD and the efficacy of anti-VEGF therapy response. Methods: Genotyping by allele-specific PCR was performed in 317 controls and 315 nAMD patients in the Russian population. Of them, 196 treatment-naive nAMD patients underwent three monthly intravitreal injections (IVIs) of aflibercept. Genotypic frequencies were compared with OCT markers of therapy effectiveness and best-corrected visual acuity (BCVA) measures. The main outcomes were the BCVA gain and decrease in central retinal thickness (CRT). Results: *MTOR*-rs1057079-C, *MTOR*-rs11121704-C and *MTOR*-rs2295080-G alleles were associated with an increased risk of nAMD. The BCVA was increased in 117 (59.7%) patients by 10 [5–20] letters, did not changed in 59 (30.1%), and was decreased in 20 (10.2%) patients. ULK1-rs3088051 was associated with BCVA change. Among patients with the TT and CT genotypes for *ULK1*-rs3088051, an improvement in visual acuity was noted in 67.6% and 53.8% of cases, while in patients with the CC genotype, an increase in BCVA was recorded in 37.5% of cases (*p* = 0.01). The decrease in CRT was associated with *SQSTM1*-rs10277 (*p* = 0.001): it was significantly higher in TT (93 [58–122] mkm) and CT (66 [30–105] mkm) carriers compared to the CC genotype (47 [24–68] mkm). Other SNPs did not show significant associations with the outcome of anti-VEGF treatment. Conclusions: *MTOR* gene polymorphisms are moderately associated with the risk of nAMD. *SQSTM1*-rs10277 and *ULK1*-rs3088051 may influence short-term response to intravitreal anti-VEGF treatment. The results suggest that autophagy could be a target for future drugs to overcome resistance to anti-VEGF therapy.

## 1. Introduction

Age-related macular degeneration (AMD) is the leading cause of irreversible late-onset blindness in developed countries. A recent meta-analysis of epidemiologic studies estimated that around 25.3% of people aged over 60 years present early or intermediate AMD, while 2.4% present late AMD [1]. Due to population ageing, a number of AMD patients is expected to increase by a third in the coming decades [2]. AMD is a progressive disease affecting the macular area due to developing pathological processes in the retinal pigment epithelium (RPE), Bruch’s membrane and choriocapillaries [3]. Because of the significant multifactorial nature and complexity of the disease, the exact mechanism of AMD pathogenesis remains uncertain. Most commonly, AMD starts in its dry form, which may progress to a wet form or neovascular AMD (nAMD) in 10–20% of cases [2]. The main symptom of nAMD is the macular neovascularization (MNV)—the pathological growth of new blood vessels into the different layers of the central retina, causing the accumulation of subretinal fluid (SRF), intraretinal fluid (IRF) and RPE detachment (PED) [4]. Without treatment, nAMD causes 90% of cases of severe vision loss [2]. Therefore, it is highly desirable to develop effective pharmacological treatment of macular degeneration [5].

The current standard of nAMD treatment is the antiangiogenic therapy targeting vascular endothelial growth factor (VEGF), which has been a successful breakthrough in the treatment of MNV. However, it requires repeated and costly intravitreal injections that cannot be postponed, as well as frequent ophthalmological examinations, making treatment expensive and time-consuming [6]. Moreover, despite its effectiveness, in some cases, there is a discrepancy between the expected clinical morphological and functional data and the obtained results: the therapeutic effect may be reduced or even absent. Up to 30% of patients show an unsatisfactory response to anti-VEGF treatment, suggesting additional factors are at work [7]. Variability in therapeutic response may be due to hereditary factors such as genetic polymorphism. Indeed, the studies indicate that in patients with nAMD, the response to anti-VEGF therapy depends on the genotype of genes in the complement system [6,8,9,10,11,12] and VEGF-related pathway [6,13,14,15]. Recently, Paterno et al. [16] found that single nucleotide polymorphisms (SNPs) of autophagy genes have been associated with nAMD and the outcomes of anti-VEGF treatment in a cohort of Finnish patients. The accumulated data indicate that genetic predisposition contributes to resistance to anti-VEGF therapy. However, genetic factors could be population-specific. Thus, studies that focused on the identification or replication of susceptibility genes in AMD development and on the response to treatments in different populations do not lose their relevance [12].

Autophagy is a lysosomal-dependent degradation process that is highly conserved and maintains cellular homeostasis by sequestering cytosolic material for degradation [17]. Dysfunctional autophagy leads to pathological accumulation of the cargo, which has been linked to a range of human diseases, including neurodegenerative diseases, infectious and autoimmune diseases, and various forms of cancer [18]. Recent studies proved the critical role of autophagy in the homeostasis of aging RPE cells [19]. Disturbances in waste clearance result in the accumulation of harmful lipid and protein aggregates, which can act as a physical barrier to intracellular transport and disrupt RPE cell function [20]. According to a number of characteristics, AMD can be attributed to ‘autophagopathies’—a class of complex human diseases whose etiology is failure in the work of the autophagy machinery, whether directly or indirectly related to an abnormal flux in autophagy, LC3-associated phagocytosis or any associated trafficking [21]. Recently, autophagy has been implicated to cause tumor resistance to antiangiogenic therapy [17], which suggests an analogous connection between autophagy and anti-VEGF intravitreal injections during AMD treatment. Considering all of the above, we assumed that genetic variability in autophagy pathway genes may influence the risk of nAMD and the response to anti-VEGF therapy.

Here, we analyzed nine SNPs in autophagy-related genes (Table 1) for the association with nAMD in a cohort of Russian patients. Further, we studied the effect of these gene variants on anatomical and functional response to aflibercept treatment. 

## 2. Materials and Methods

### 2.1. Study Participants

This study was conducted in accordance with the ethical principles of the Declaration of Helsinki and the National Standard for Good Clinical Practice and was approved by the Institutional Review Board at the Institute of Cytology and Genetics SB RAS. The subjects were informed and had provided written consent to the collection and scientific use of the specimen prior to the procedure. The AMD group consisted of 315 patients (98 (31%) men and 217 (69%) women with a mean age of 71.5 ± 8.6 years) diagnosed with nAMD at the Department of Ophthalmology of the Novosibirsk Regional Clinical Hospital. The control group comprised 317 subjects (103 (32%) men and 214 (68%) women with a mean age of 66.5 ± 7.5 years), undergoing routine cataract surgery without a history of AMD and macular changes such as drusen or pigment abnormalities [22]. A complete ophthalmological examination was performed, including visometry, biomicroscopy, ophthalmoscopy, and optical coherence tomography (OCT). Exclusion criteria from the study were active neovascularization in the periphery of the retina and in the anterior segment of the eye, a history of laser photocoagulation, medical intravitreal therapy in history, spherical equivalent more than ±6.0 diopters, uveitis, geographic atrophy, surgical interventions on the vitreous body, the presence of signs of intraocular inflammation, pathology of the vitreomacular interface with traction component, polypoidal choroidal vasculopathy or any other confounding retinopathies [12,22].

### 2.2. Optical Coherence Tomography Study

The subset OCT study included 196 treatment-naive patients diagnosed with nAMD. After being diagnosed, all patients began to receive anti-VEGF therapy. Intravitreal injections (IVIs) of aflibercept (Regeneron, Munich, Germany) (0.05 mL (2 mg)) were administered according to the standard method in the operating room after local epibulbar anesthesia with an alkaline solution (Alcon, Fort Worth, TX, USA) through a 31 G needle at least 3 mm from the limbus. Three successive injections were performed with an interval of 4 weeks. To assess the effectiveness of therapy, a clinical and instrumental examination of patients was performed by OCT (Cirrus HD-OCT, Humphrey Zeiss, Inc., Jena, Germany) and visometry, with the determination of best corrected visual acuity (BCVA) at baseline and after three IVIs [22]. The following parameters were assessed: type of macular neovascularization (MNV), BCVA, central retinal thickness (CRT), height of pigment epithelium detachment (PED), height of subretinal fluid (SRF) and the presence of intraretinal fluid (IRF). BCVA was estimated using a letter count on the Early Treatment of Diabetic Retinopathy Study (ETDRS) chart.

### 2.3. DNA Isolation and Genotyping

Peripheral venous blood was collected in vacutainers with EDTA for DNA analysis at baseline visit. Genomic DNA was isolated by DNA Blood Kit (Biolabmix, Novosibirsk, Russia) according to the manufacturer’s protocol. Genotyping was carried out with TaqMan-based allelic discrimination assays. Primers and probes were designed using Primer-Blast (https://www.ncbi.nlm.nih.gov/tools/primer-blast/ (accessed on 1 January 2021) and Oligo Analyzer (version 1.0.3) (Table 2). LNA (locked nuckeic acid) modifications were used to obtain the optimal melting temperature in probes. PCR was performed in 20 μL reaction volume containing 20 ng of genomic DNA, BioMaster HS-qPCR (2×) buffer (Biolabmix), 0.3 mM primers and 0.1 mM FAM/VIC-conjugated probes. PCR thermal cycling conditions were as follows: denaturation for 3 min at 95 °C followed by 35 cycles, including denaturation at 95 °C for 10 s, primer annealing and subsequent elongation at 60 °C for 30 s. Amplification was conducted using CFX96 Thermal Cycler (Bio-Rad, Hercules, CA, USA). The PCR data were processed using «Bio-Rad CFX Manager 3.1» software, Russian Edition #1845028, Novosibirsk, Russia. To verify the results of allelic discrimination, Sanger sequencing was used in samples from different genotypes on an ABI 3500 DNA sequencer (Thermo Fisher Scientific, Waltham, MA, USA) by means of the BigDye Terminator v3.1 Cycle Sequencing Kit (Thermo Fisher Scientific, Waltham, MA, USA).

### 2.4. Statistical Analysis

An analysis of the comparisons of frequencies of genotypes between AMD and control groups was performed using the chi-square test. SNPs with genotype frequencies that differ significantly between groups (*p*-value < 0.05, χ^2^ < 5.991) were selected for further analysis. To evaluate the effects of these SNPs, odds ratios (ORs) and 95% confidence intervals (CIs) were calculated using a logistic regression analysis adjusted for sex and age adopting codominant, dominant, recessive, overdominant and additive models of inheritance using SNPstats [23]. The significance threshold after the implementation of Bonferroni correction for multiple testing was set on *p* = 0.05/15 = 0.003. 

Statistical analysis of association between SNPs and OCT markers was performed using StatTech v. 3.1.10 (Developer—StatTech LLC, Russia). Quantitative variables were assessed for normality using the Kolmogorov–Smirnov test. Quantitative variables following a normal distribution were described using mean (M) and standard deviation (SD). Quantitative variables following non-normal distribution were described using median (Me) and lower and upper quartiles (Q1–Q3). Categorical data are shown as absolute values (percentage). Comparisons of three groups on a quantitative variable whose distribution differed from normal were made using the Kruskal–Wallis test and Dunn’s criterion with Holm correction as a post hoc method. Comparison of frequencies in the analysis of multifield contingency tables was performed using Pearson’s chi-square test (for expected values greater than 10). Wilcoxon test was used for comparison of quantitative variable following non-normal distribution between two matched samples. Comparison of binary variables in two paired samples was performed using McNemar test. The differences were considered significant at *p* < 0.05. 

## 3. Results

### 3.1. Association with Risk of nAMD

The genotypes of SNPs of the mTOR (rs1064261, rs1057079, rs11121704, rs2295080), SQSTM1 (rs10277), Ulk1 (rs11246867, rs3088051), MAP1LC3A (rs73105013) and Atg5 (rs573775) genes were determined in the AMD and in the control group (Table 3). The allele frequencies calculated for ethnic Russians in our study were close or similar to the corresponding frequencies in European populations reported by the 1000 Genomes Project. The genotype frequencies of rs1057079, rs11121704 and rs2295080 of the mTOR gene differ significantly between groups (*p*-value < 0.05, χ^2^ < 5.991). These SNPs were selected for an logistic regression analysis adjusted for sex and age to evaluate the odds ratios (ORs) and 95% confidence intervals (CIs) (Table 4).

The MTOR rs1057079 C/T genotype was associated with increased odds of nAMD risk under the codominant (OR = 1.85; CI: 1.31–2.61; *p* = 0.0018) and overdominant (OR = 1.83; CI: 1.31–2.56; *p* = 0.00004) models, and C/T-C/C genotypes were associated with 1.7-fold (OR = 1.7; CI: 1.22–2.36; *p* = 0.0017) increased odds of nAMD according to the dominant model (Table 4). Each C allele increases the odds of developing nAMD by 1.34-fold under the additive model (OR = 1.54; CI: 1.07–2.20; *p* = 0.017). According to Akaike’s information criterion (AIC), the overdominant model was preferable.

The MTOR rs11121704 C/T genotype was associated with increased odds of developing AMD under the codominant (OR = 1.74; CI: 1.23–2.46; *p* = 0.0067) and overdominant (OR = 1.63; CI: 1.17–2.27; *p* = 0.0038) models, and C/T+C/C genotypes were associated with 1.69-fold (OR = 1.69; CI: 1.21–2.35; *p* = 0.0018) increased odds of AMD according to the dominant model. Each C allele increases the odds of developing AMD by 1.4-fold under the additive model (OR = 1.4; CI: 1.09–1.81; *p* = 0.0094). According to AIC, the dominant model was preferable.

The MTOR rs2295080 G/T genotype was associated with increased odds of a risk of nAMD under the codominant (OR = 1.86; CI: 1.31–2.64; *p* = 0.0021) and overdominant (OR = 1.77; CI: 1.27–2.47; *p* = 0.0007) models, and G/T+G/G genotypes were associated with 1.74-fold (OR = 1.74; CI: 1.24–2.42; *p* = 0.0011) increased odds of AMD according to the dominant model. Each G allele increases the odds of developing AMD by 1.35-fold under the additive model (OR = 1.35; CI: 1.05–1.73; *p* = 0.019). According to AIC, the overdominant model was preferable.

Bonferroni correction was performed to reduce type I error in multiple testing, and a significant threshold was set at 0.05/15 = 0.003 for genotype analyses. After that, only the codominant, dominant and overdominant models for rs1057079; the dominant model for rs11121704; and the dominant and overdominant models for rs2295080 were still valid.

The overdominant model of inheritance may be explained with the fact that all components, including mTOR, are dimerized in the mTORC1 and mTORC2 complexes. Noncoding substitutions in the MTOR gene can lead to changes in mRNA stability, interaction with transcription factors, and the rate of protein synthesis, and the simultaneous presence of two heterogeneous protein populations in heterozygous cells can affect the structure or concentration of an efficient homodimer in the cell.

An analysis of linkage disequilibrium (LD) shows strong non-random association of all variants in the MTOR gene (spanning region of 117.5 kb) with D’ > 0.867. The GCCG haplotype, corresponding to the minor alleles of rs1064261, rs1057079, rs11121704 and rs2295080, respectively, was associated with 1.45-fold (OR = 1.54; CI: 1.09–1.92; *p* = 0.01) increased odds of nAMD compared with the most frequent ATTT haplotype.

No evidence of association with nAMD risk was observed for the other SNPs.

### 3.2. Association between SNPs and OCT Markers

In total, 196 patients with treatment-naive nAMD were included in the OCT study. The mean age of the study population was 71 ± 9 years, of which there were 60 (30.6%) males and 136 (69.4%) females. The OCT characteristics of the study participants are shown in Table 5. According to the type of MNV, MNV type 1 was diagnosed in 93 (47.4%) eyes, MNV type 2 was diagnosed in 94 (48%) eyes and MNV type 3 was diagnosed in 9 (4.6%) eyes. The median baseline PED height was 126 µm (interquartile range (IQR) 89 µm to 190 µm), mean baseline CRT was 316 µm (IQR 271 µm to 372 µm) and median baseline SRF height was 126 µm (IQR 89 µm to 190 µm). IRF was seen in 140 eyes (71.4%). According to OCT, a significant BCVA gain and significant decreases in CRT, PED and SRF height were achieved in patients after three loading IVIs (Table 5).

The decrease in CRT was associated with SQSTM1-rs10277 (*p* = 0.001): it was significantly higher in TT (93 [58–122] µm) and CT (66 [30–105] µm) carriers compared with the CC genotype (47 [24–68] µm) (Table 6).

**Figure 1:**
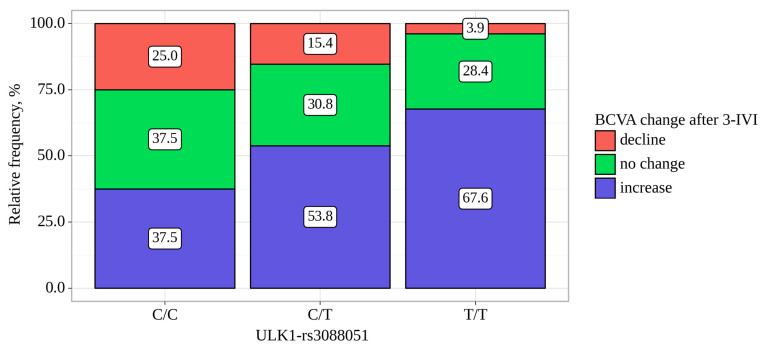
Analysis of BCVA change after three IVIs depending on *ULK1*-rs3088051.

The BCVA was increased in 117 (59.7%) patients by 10 [5–20] letters, did not changed in 59 (30.1%) and was decreased in 20 (10.2%) patients. ULK1-rs3088051 was associated with BCVA change. Among patients with the TT and CT genotypes for the ULK1-rs3088051, an improvement in visual acuity was noted in 67.6% and 53.8% of cases, while in patients with the CC genotype, an increase in BCVA was recorded in 37.5% of cases (*p* = 0.01) (Table 7, Figure 1). Moreover, in carriers of the CC genotype, the median BCVA change was the smallest—0 letters (IQR −0 to −6 letters)—while in CT and TT carriers, it was 5 letters (IQR 0 to 12 letters) and 5 letters (IQR 0 to 16 letters), respectfully (*p* = 0.008, Table 7).

Other SNPs did not show significant associations with the outcome of anti-VEGF treatment.

## 4. Discussion

AMD is a complex degenerative disease of the retina with multiple risk-modifying factors, including aging, genetics and diet. The progression of AMD is initially characterized by atrophic alterations in the RPE, as well as the formation of lysosomal lipofuscin and extracellular drusen deposits [21]. Autophagy is a catabolic process indispensable for retinal cell homeostasis. The role of autophagy in AMD pathology and treatment is steadily emerging [24]. Although anti-VEGF therapy has achieved a good therapeutic effect in nAMD, the recurrence of MNV is inevitable, which increases the cost of treatment [25]. Therefore, it is especially important to uncover the mechanisms of the processes that control the development of MNV and to reduce its recurrence. According to the latest data [17,18,21], autophagy may be one of these controlling processes. In particular, attention has been drawn to research in the field of cancer therapy, where, as in nAMD, anti-VEGF drugs are used [26]. Recent studies have shown that autophagy plays a key role in the mechanism of formation of tumor resistance to antiangiogenic therapy [17].

As well as in tumor treatment, autophagy may be responsible for resistance development to anti-VEGF therapy during nAMD treatment. Recently, the in vitro study showed that ranibizumab and conbercept can trigger the autophagy of vascular endothelial cells while aflibercept can inhibit it. The mechanism of autophagy activation was related to the activation of the p53/DRAM pathway [25]. 

In this study, we showed that regulatory SNPs (rs1057079, rs11121704 and rs2295080) in the MTOR gene are moderately associated with a risk of nAMD in the Russian population. The kinase mTOR (mechanistic Target of Rapamycin) affects the most fundamental cellular processes. It is essential for cellular and organismal physiology, and its dysregulation is frequently associated with human aging and age-related diseases. As the PI3K/AKT/mTOR signaling pathway’s key player, mTOR is essential for the sensing of cellular energy, oxygen and nutrients [27]. The most significant stimulus that controls mTORC1 activity is nutrient deficiency. Actually, mTORC1 serves as an amino acid molecular sensor, connecting cellular demand with dietary supply [28]. Acting as a crucial upstream regulator of autophagy, mTOR inhibits the ULK1-ATG13-RB1CC1/FIP200 complex on the initial step of autophagy. Recently, it was shown that the mTOR pathway is partially responsible for the mitochondrial damage caused by complement factor H protein loss in RPE cells [29]. Previously, a similar link between SNPs (rs1057079, rs11121704 and rs2295080) in the MTOR gene and wet AMD was found in the Finnish population [16]. It is known that pharmacogenetic association may differ by ethnicity if the allele distribution of a candidate polymorphism varies among populations. As a result, the studies aimed at identifying or replicating genes for susceptibility to AMD risk and response to treatment in various populations continue to be relevant [12].

The main aim of pharmacogenetic studies is to identify the patients who could benefit the most from treatment. Our study links polymorphisms in autophagy-related genes to anti-VEGF treatment response. We showed that Sqstm1-rs10277 and Ulk1-rs3088051 influence short-term response to aflibercept therapy. The results are consistent with the study by Paterno et al. [16], where a correlation was also found between these polymorphisms and the response to antiangiogenic therapy.

The members of the ULK (Unc-51-like kinase) family of proteins are the orthologues of the yeast Atg1, a serine/threonine protein kinase essential for autophagy initiation [30]. ULK1 or ULK2 are part of a protein complex that is responsible for driving autophagy initiation upon autophagy-inducing stimuli [31]. Moreover, the ULK1/2 protein complex carries other different autophagy-related functions, such as ATG9-vesicle recruitment or regulation of ATG4B activity, and contributes to regulating the mitophagy and degradation of protein aggregates [32]. Several pathogenic variants of the ULK1/2 kinase complex have been identified [30]. Polymorphisms in members of the ULK1/2 kinase complex have been associated with a variety of pathologies related to immune system dysfunction: Crohn’s disease susceptibility [33], tuberculosis [34] and ankylosing spondylitis [35].

The SQSTM1 gene codes a p62 protein—a multifunctional protein important for protein aggregate degradation, mitophagy and the engulfment of intracellular pathogens by autophagosomes [36] and the most studied ubiquitin-binding selective autophagy receptor (SAR). SARs mediate the recognition and engulfment of specific cargo in autophagosomes by simultaneously binding both to the target molecules and to the ATG8 proteins conjugated on the concave side of the autophagosomal membrane [37]. The dysregulation of SAR function plays a role in the pathogenesis of a wide variety of diseases, with most pathogenic variants being identified in the SQSTM1 gene [30]. Nucleotide changes on SQSTM1 are known to contribute to the origin of neurological alterations [38], implicated in the pathogenesis of amyotrophic lateral sclerosis [39] and frontotemporal dementia [40].

Obviously, angiogenesis, including VEGF-dependent pathological vascular growth, and autophagy are closely related processes. Our results fit into the concept recently proposed by the Grosjean et al. [21]. The authors of this model proposed the term “autophagopathy” to encompass a class of genetic diseases whose etiology is associated with a defect in the autophagy machinery, whether directly related to abnormal autophagic flux, LC3-associated phagocytosis or any concomitant process of cellular debris removal. This model suggests that neither genetic variants of autophagy nor environmental exposures alone cause disease. However, SNPs that result in low-level autophagy may alter the cell’s ability to detoxify damaged organelles when exposed to environmental factors. As a consequence, regulatory autophagy-related SNPs will predispose individuals to develop broad range of diseases (such as cancer, infections, neurodegenerative, cardiovascular, metabolic and inflammatory diseases) only when exposed to this specific environmental risk, the nature of which will determine the affected organ and disease [21]. Therefore, it will be critical to recognize these autophagy-related SNP carriers to aid disease screening, prevention and precision treatment programs to rapidly alleviate comorbid complications [21]. Similarly, it can be assumed that the carriage of certain variants of autophagy genes, which determine the characteristics of the autophagy apparatus under stress, can influence the success of antiangiogenic therapy, as we and Paterno et al. [16] have shown for nAMD. Therefore, autophagy SNP genotyping may be useful for investigating treatment response to other diseases that use an antiangiogenic protocol, such as diabetic macular edema, retinopathy of prematurity and various types of cancer. To our knowledge, there are no ongoing clinical trials targeted at the autophagy pathway in nAMD. Our results suggest that such trials may be relevant and that autophagy could be a target for future drugs to overcome resistance to anti-VEGF therapy.

A limitation of our study is the relatively small sample size of the patient cohort, which reduces the statistical power available to detect statistically significant associations. Another limitation of our study is the use of patients with cataracts as a control group due to the inability to recruit healthy elderly people. Also, we studied only short-term response. On the other hand, a significant time point for the prediction of long-term functional outcomes may be the period after the three IVIs associated with the loading phase [41]. The strength of this study is the inclusion of patients who had never received treatment, thereby eliminating any potential influence of a retreatment regimen.

## 5. Conclusions

Our study provides further insight into the pharmacogenetics of the clinical response of nAMD to anti-VEGF therapy by identifying an association between autophagy-related gene polymorphisms and treatment response outcome. We confirmed the previously found effects of polymorphisms in autophagy genes on both nAMD risk and anti-VEGF response in a larger and independent cohort of patients. Based on our results, (1) variants in the mTOR gene are associated with nAMD risk, and (2) Sqstm1-rs10277 and Ulk1-rs3088051 could be considered as biomarkers of response to anti-VEGF treatment in nAMD patients. However, to uncover the molecular mechanisms mediating the influence of autophagy on VEGF pathway in AMD vulnerable eye tissues, further studies with the inclusion of in vivo and in vitro model systems are necessary.

## Figures and Tables

**Table 1:** SNPs analyzed in the study.

Gene	SNP	Location/Consequence	Position	Minor Allele	MAF
*ATG5*	rs573775	Intron variant	chr6:106316991	A	0.27
*MAP1LC3A*	rs73105013	Intron variant	chr20:34557008	C	0.08
*MTOR*	rs1064261	Missense variant	chr1:11228701	G	0.28
	rs1057079	Synonymous variant	chr1:11145001	C	0.26
	rs11121704	Intron variant	chr1:11233902	C	0.28
	rs2295080	Upstream variant	chr1:11262571	G	0.31
*SQSTM1*	rs10277	3 Prime UTR Variant	chr5:179837731	T	0.48
*ULK1*	rs11246867	Upstream variant	chr12:131893472	A	0.06
	rs3088051	3 Prime UTR Variant	chr12:131922463	C	0.3

SNP: single nucleotide polymorphism. MAF: minor allele frequency in European populations according to 1000 Genomes.

**Table 2:** Primers/probes sequences.

rsID	Gene Name	Sequence
rs573775	*ATG5*	Forward 5′-CCCTACCTAGTATGCTCCTC-3′
		Reverse 5′-AAAAGCCATGTCCTTATGCC-3′
		5′-FAM-CCTCTGGCCCCAGTGAAACAG-BHQ1-3′
		5′-VIC-CTCTGGCCCCA[+A]TGAAACAGT-BHQ1-3′
rs1064261	*MTOR*	Forward 5′-AAGGATTGGGGTTTGAGGTA-3′
		Reverse 5′-GACCAGTCACTCTCTCATCA-3′
		5′-FAM-CACGTTCCTTAA[+C]GTCATTCGA-BHQ1-3′
		5′-VIC-CACGTTCCTTAA[+T]GTCATTCGA-BHQ1-3′
rs1057079	*MTOR*	Forward 5′-GCAGCCTGTAAGTTCTCAAT-3′
		Reverse 5′-CCCAAGGGTTGTTTCTCTTC-3′
		5′-FAM-CTCCTGCCATCGCAGTTAATTCA-BHQ1-3′
		5′-VIC-CTCCTGCCAT[+T]GCAGTTAATT-BHQ1-3′
rs11121704	*MTOR*	Forward 5′-TTTTTCCTCATTTTGGGCGA-3′
		Reverse 5′-TATCAGTTGCAGGAAAGTGC-3′
		5′-FAM-CAGGCACATCATCGCAGATGTTT-BHQ1-3′
		5′-VIC-CAGGCACATCATCACAGATGTTTG-BHQ1-3′
rs2295080	*MTOR*	Forward 5′-TTCCCCGCTGTCCTCTA-3′
		Reverse 5′-GCCTGTTTTTCAGTCCATCT-3′
		5′-FAM-CCTCAGGGCTGGGAACCC-BHQ1-3′
		5′-VIC-CCTCAGGG[+A]TGGGAACCCTC-BHQ1-3′
rs73105013	*MAP1LC3A*	Forward 5′-CAGCCTTAAAAACAAAAACCCT-3′
		Reverse 5′-ATGGAAGGCAGAAAGGGAGA-3′
		5′-FAM-CTTATCCCCAG[+T]GTCTTCTGC-BHQ1-3′
		5′-VIC-CTTATCCCCAG[+C]GTCTTCTGC-BHQ1-3′
rs10277	*SQSTM1*	Forward 5′-GTCCCTCTGAAGAGACCTTG-3′
		Reverse 5′-CTGGGAAGGAGCTATGGAG-3′
		5′-FAM-AGGACAAAT[+T]GCGCCCAT-BHQ1-3′
		5′-VIC-CAGGACAAATCGCGCCCATT-BHQ1-3′
rs11246867	*ULK1*	Forward 5′-GTACGGTGAACAGCACTAAC-3′
		Reverse 5′-CAGCCAAAAGAGCCCG-3′
		5′-FAM-CAGCCAACAG[+C]GATTGCTCT-BHQ1-3′
		5′-VIC-CAGCCAACAG[+T]GATTGCTCT-BHQ1-3′
rs3088051	*ULK1*	Forward 5′-GGAAGCAGATGAGGGGAATA-3′
		Reverse 5′-CTCTCTGCAGATGCCCTC-3′
		5′-FAM-CAGTCAGTTT[+T]GATGTCAGCTC-BHQ1-3′
		5′-VIC-CAGTCAGTTT[+C]GATGTCAGCTC-BHQ1-3′

[+X]—LNA modifications.

**Table 3:** Allele and genotype frequencies in the nAMD and control groups in a Russian cohort.

SNP	Genotype/Allele	Control	AMD	*p*-Value, χ^2^
rs573775 *ATG5*	A/A	24 (8%)	26 (8%)	*p* = 0.838, 0.354
	G/A	145 (46%)	137 (43%)	
	G/G	148 (47%)	152 (48%)	
	MAF	0.3	0.3	
rs1064261 *MTOR*	A/A	150 (47%)	125 (40%)	*p* = 0.093, 4.757
	A/G	130 (41%)	156 (50%)	
	G/G	37 (12%)	34 (11%)	
	MAF	0.32	0.36	
rs1057079 *MTOR*	C/C	28 (9%)	21 (7%)	*p* = 0.002, 12.563
	T/C	109 (34%)	152 (48%)	
	T/T	180 (57%)	142 (45%)	
	MAF	0.26	0.31	
rs11121704 *MTOR*	C/C	28 (9%)	29 (9%)	*p* = 0.006, 10.324
	T/C	121 (38%%)	158 (50%)	
	T/T	168 (53%)	128 (41%)	
	MAF	0.28	0.34	
rs2295080 *MTOR*	G/G	36 (11%)	31(10%)	*p* = 0.002, 12.562
	T/G	123 (39%)	166 (53%)	
	T/T	158 (5%)	118 (37%)	
	MAF	0.31	0.36	
rs73105013 *MAP1LC3A*	C/C	2 (0.6%)	0 (0%)	*p* = 0.169, 3.557
	T/C	41 (13%)	52 (17%)	
	T/T	273 (86%)	261 (83%)	
	MAF	0.07	0.08	
rs10277 *SQSTM1*	C/C	118 (37%)	106 (34%)	*p* = 0.385, 1.913
	C/T	157 (50%)	156 (50%)	
	T/T	42 (13%)	53 (17%)	
	MAF	0.38	0.42	
rs11246867 *ULK1*	A/A	2 (0.6%)	1 (0.3%)	*p* = 0.574, 1.113
	G/A	33 (10%)	40 (13%)	
	G/G	282 (89%)	274 (87%)	
	MAF	0.06	0.07	
rs3088051 *ULK1*	C/C	32 (10%)	27 (9%)	*p* = 0.579, 1.096
	T/C	121 (38%)	132 (42%)	
	T/T	164 (52%)	156 (50%)	
	MAF	0.29	0.3	

χ^2^—chi-square test. The critical value of χ^2^ at the significance level *p* < 0.05 is 5.991. The null hypothesis is rejected if χ^2^ > 5.99.

**Table 4:** Association of the *MTOR* polymorphisms with the risk of nAMD in a Russian cohort.

SNP	Model of Inheritance	OR (95% CI) Adjusted for Sex and Age by Logistic Regression	*p*-Value	AIC
rs1057079 *MTOR*	Codominant:		0.0018	814.5
	C/T vs. T/T	1.85 (1.31–2.62)		
	C/C vs. T/T	1.07 (0.57–2.03)		
	Dominant: C/T-C/C vs. T/T	1.70 (1.22–2.36)	0.0017	815.2
	Overdominant: C/T vs. C/C-T/T	1.83 (1.31–2.56)	0.0004	812.5
	Recessive: C/C vs. C/T-T/T	0.82 (0.44–1.51)	0.51	824.6
	Additive	1.34 (1.03–1.74)	0.028	820.2
rs11121704 *MTOR*	Codominant:		0.0067	817
	C/T vs. T/T	1.74 (1.23–2.46)		
	C/C vs. T/T	1.47 (0.81–2.68)		
	Dominant: C/T-C/C vs. T/T	1.69 (1.21–2.35)	0.0018	815.3
	Overdominant: C/T vs. C/C-T/T	1.63 (1.17–2.27)	0.0038	816.7
	Recessive: C/C vs. C/T-T/T	1.13 (0.64–2.00)	0.68	824.9
	Additive	1.40 (1.09–1.81)	0.0094	818.3
rs2295080 *MTOR*	Codominant:		0.0021	814.8
	G/T vs. T/T	1.86 (1.31–2.64)		
	G/G vs. T/T	1.29 (0.73–2.26)		
	Dominant: G/T-G/G vs. T/T	1.74 (1.24–2.42)	0.0011	814.4
	Overdominant: G/T vs. G/G-T/T	1.77 (1.27–2.47)	0.0007	813.5
	Recessive: G/G vs. G/T-T/T	0.93 (0.55–1.59)	0.8	825
	Additive	1.35 (1.05–1.73)	0.019	819.6

OR: odds ratio; CI: confidence interval; AIC: Akaike’s information criterion. The significance threshold taking into account multiple comparisons is *p* = 0.003.

**Table 5:** Characterization of functional and anatomical parameters of the retina in patients with nAMD.

	Baseline	3 IVIs	*p*-Value
BCVA, letters	48 ± 22	55 ± 21	<0.001 ^a^
CRT, mkm	316 [271–372]	246 [218–289]	<0.001 ^a^
PED height, mkm	126 [89–190]	46 [21–96]	<0.001 ^a^
SRF height, mkm	56 [29–90]	22 [0–44]	<0.001 ^a^
IRF, abs. (%)	140 (71.4)	89 (45.4)	<0.001 ^b^

BCVA—best corrected visual acuity; CRT—central retinal thickness; PED—detachment of the pigment epithelium; SRF—subretinal fluid; IRF—intraretinal fluid. Mean ± sd or Median (Q1–Q3). Applied methods for matched samples: ^a^ Wilcoxon test, ^b^ McNemar test.

**Table 6:** Analysis of decrease in CRT depending on *SQSTM1*-rs10277.

SNP	Genotype	Decrease in CRT, µm			*p*-Value
		Me	Q1–Q3	n	
rs10277 *SQSTM1*	C/C	47	24–68	63	0.001 *p* _C/T–C/C_ = 0.014 *p* _T/T–C/C_ = 0.002
	C/T	66	30–105	100	
	T/T	93	58–122	33	

Applied method: Kruskal–Wallis test and Dunn’s criterion with Holm correction as a post hoc method.

**Table 7:** Analysis of BCVA change after three IVIs depending on *ULK1*-rs3088051.

*ULK1*-rs3088051	BCVA Change, abs. (%).			*p*-Value, Pearson’s Chi-Square
	Decline	no Change	Increase	
C/C	4 (25.0)	6 (37.5)	6 (37.5)	0.013 *p* _C/C–T/T_ = 0.010 *p* _C/T–T/T_ = 0.037
C/T	12 (15.4)	24 (30.8)	42 (53.8)	
T/T	4 (3.9)	29 (28.4)	69 (67.6)	
*ULK1*-rs3088051	BCVA change, letters.			*p*-value, Kruskal–Wallis
	Me	Q1–Q3	n	
C/C	0	−0–6	16	0.008 *p* _T/T–C/C_ = 0.022
C/T	5	0–12	78	
T/T	5	0–16	102	

## Data Availability

The datasets are available from the corresponding author on request.
